# Monthly Record of Deaths in the City of Buffalo

**Published:** 1856-06

**Authors:** C. L. Dayton

**Affiliations:** Health Physician


ART. VIII.—*Report of Mortality in Buffalo for the month of April* 1856. By C. L. DAYTON, M. D., Health Physician.

| DISEASES. | No. | Males. | Females. |
|---|---|---|---|
| Asphyxia, | 1 | 1 | |
| " Immersorum, | 3 | 2 | 1 |
| Apoplexia, | 2 | 1 | 1 |
| Bronchitis, | 2 | 2 | |
| " Capillary, | 1 | 1 | |
| Cancer, | 1 | | 1 |
| Chorea, | 1 | 1 | |
| Cynanche Trachealis, | 2 | 1 | 1 |
| Constipatio Alvi, | 1 | 1 | |
| Diarrhœa, | 2 | 1 | 1 |
| Debilitas Senilis, | 3 | 3 | |
| Dissipatio, | 1 | | 1 |
| Enteritis, | 1 | 1 | |
| Erysipelas, | 1 | 1 | |
| Encephalitis, | 2 | 1 | 1 |
| Eclampsia, | 16 | 8 | 8 |
| Febris Typhoid, | 5 | 3 | 2 |
| " Puerperarum, | 1 | | 1 |
| " Intermittent, (Homœopath) | 1 | 1 | |
| " Scarlatina, | 2 | 1 | 1 |
| Fracture of Scull, | 1 | 1 | |
| Gastro Enteritis Chronica, | 1 | | 1 |
| Hepatic Abscess, | 1 | 1 | |
| Hyperæmia Pulmonalis, | 1 | 1 | |
| " Cerebralis, | 1 | | 1 |
| Hydrops, | 3 | 2 | 1 |
| " Renalis, | 1 | | 2 |
| " Cerebri, | 2 | | 2 |
| " Pericardii, | 1 | | 1 |
| Icteritis, | 1 | 1 | |
| Ignotus, | 9 | 6 | 3 |
| Malignant Pustule, | 1 | | |
| Medullary Sarcoma, | 1 | 1 | |
| Marasmus, | 4 | 2 | 1 |
| Morbus Cordis, | 1 | 1 | |
| " Hepatitis, | 1 | | 1 |

### REGISTER OF MORTALITY—Continued.

| DISEASES. | No. | Males. | Females. |
|---|---|---|---|
| Pertussis, | 1 | 1 | |
| Paralysis, | 2 | 1 | 1 |
| Pneumonitis, | 5 | 4 | 1 |
| " Double, | 1 | | 1 |
| " Typhoides, | 1 | 1 | |
| Still-born, | 5 | 2 | 2 |
| Tuberculosis Pulmonalis, | 18 | 10 | 8 |
| Tetanus, | 1 | 1 | |
| Total, | 113 | | |

### SEXES.

| | |
|---|---|
| Males, | 66 |
| Females, | 44 |
| Sex not given, | 3 |
| Total, | 113 |

### AGES.

| | |
|---|---|
| Still-born, | 5 |
| 1 day, | 0 |
| Between 1 day and 30 days, | 4 |
| " 1 month and 6 months, | 10 |
| " 6 months and 12 " | 9 |
| " 1 year " 3 years, | 15 |
| " 3 " " 5 " | 2 |
| " 5 " " 10 " | 5 |
| Total number under 10 years, | 50 |
| Between 10 years and 20 years, | 6 |
| " 20 " " 30 " | 18 |
| " 30 " " 40 " | 14 |
| " 40 " " 50 " | 10 |
| " 50 " " 60 " | 4 |
| " 60 " " 70 " | 7 |
| " 70 " " 80 " | 1 |
| " 80 " " 90 " | 1 |
| Total number over 10 years, | 61 |

Ages not given, .........2. Total, ......... 113

### NATIVITIES.

| | |
|---|---|
| American, (white, 51) colored, 1, | 52 |
| German, | 29 |
| Irish, | 14 |
| English, | 3 |
| French, | 4 |
| Prussian, | 1 |
| Holland, | 1 |
| Scotland, | 1 |
| Country not given, | 7 |
| Poland, | 1 |
| Total, | 113 |

